# Astroviruses use mucus secretion as a novel form of viral egress

**DOI:** 10.1371/journal.ppat.1014683

**Published:** 2026-10-09

**Authors:** Natalie Pedicino, Melanie G. Kirkpatrick, Michelle Ann Pablo, Jacob Gulman, Nicole Heredia-Osuna, Valerie Cortez

**Affiliations:** Department of Molecular, Cell and Developmental Biology, University of California, Santa Cruz, California, United States of America; US Food and Drug Administration, UNITED STATES OF AMERICA

## Abstract

Viruses rely on coordinated host pathways to exit the cell and propagate infection. We have come to appreciate that non-enveloped enteric viruses can use both lytic and non-lytic strategies to exit host cells. However, it remains unclear how astroviruses, a major causative agent of pediatric diarrhea, facilitate this final step in their replication cycle. Because astroviruses infect small intestinal goblet cells, we investigated whether mucus secretion could enable a pathway of non-lytic viral egress. Using the murine astrovirus model, we found that virus release co-occurred almost exclusively with mucus secretion from goblet cells. At the tissue level, mucus secretion also correlated with infection kinetics, characterized by a return to baseline secretion during viral clearance. Further, disruption of mucin biosynthesis reduced viral shedding, and stimulating mucus secretion with carbachol increased viral release. To examine this pathway with human astrovirus, we established a new infection model in LS174T goblet cells. We observed that human astrovirus colocalized with MUC2 within the cell and with purified mucin granules. Stimulating cells with carbachol enhanced mucus and virus release, which could be blocked by the addition of a muscarinic receptor antagonist. Further, we found that blocking intracellular calcium, which drives mucin granule exocytosis, also reduced virus release. Together, these findings demonstrate that mucus secretion is a novel form of non-lytic enteric viral egress, which deepens our understanding of the astrovirus replication cycle and may hold clues to understanding its pathogenesis.

## Author summary

Astroviruses cause pediatric diarrhea worldwide, yet we understand little about how they replicate inside a cell and cause disease. Human and murine astroviruses infect goblet cells, which are the main mucus producers in the intestine. Here, we use both mouse models and human cell culture models to examine whether mucus secretion enables a pathway for astrovirus to exit from goblet cells and spread. When we blocked mucus secretion, we observed reduced virus released. Conversely, when we stimulated mucus secretion, we observed increased virus release. We also found that subcellular structures that package mucus co-isolated with virus particles. This discovery identifies a unique exit strategy for an enteric virus that allows it to spread without having to bust open and kill its host cell. Understanding this host-virus interaction during the replication cycle can provide new insights into pathogenesis and potentially new targets for treatments strategies that prevent astrovirus spread.

## Introduction

Viruses rely on coordinated host cell pathways to complete their replication cycle, resulting in release of nascent viral particles and propagation of the infection. Non-enveloped and enveloped viruses utilize different means to exit the cell, with non-enveloped viruses relying on lytic (e.g., apoptosis, necrosis) and non-lytic (e.g., exosomes, microvesicles) forms of virus release [1]. By studying how viruses exit a host cell, we can deepen our understanding of viral pathogenesis and provide insights into replication, transmission, and disease progression.

Enteric viruses, including rotavirus, norovirus, and astrovirus, are leading causes of diarrhea that replicate in the intestinal epithelium but use different mechanisms of egress. Rotavirus has been shown to use a lytic means of egress in vitro, although non-lytic vesicle-mediated pathways have also been described [2–4]. In murine models, infection results in villus atrophy and epithelial destruction [5,6]. Likewise, norovirus, has been shown to use both lytic and non-lytic forms of release [2,7,8]. In contrast, research to date indicates that astrovirus uses an unclassified form of non-lytic release. Astroviruses are non-enveloped, positive-sense single-stranded RNA viruses. Their ~7 kb genome is organized into three main open reading frames (ORFs): ORF1a and ORF1b encode non-structural proteins involved in replication and polyprotein processing, while ORF2 encodes the structural capsid precursor [9]. While the specific processes driving astrovirus egress are not yet defined, a subset of astroviruses express the viroporin, XP, which promotes replication during virus assembly and/or release [10]. Other enteric viruses also utilize specialized proteins to facilitate egress. For example, rotavirus NSP4 triggers calcium-dependent release via necroptosis [11], and norovirus NS3 facilitates egress via its pore-forming domain that causes mitochondrial dysfunction [12], while NS1/2 promotes spread via apoptosis [13]. Identifying the astrovirus-host interactions that facilitate non-lytic exit from the cell is critical to understanding its replication cycle. Astroviruses are a significant, but understudied, causative agent of viral diarrhea in young children worldwide, second only to rotavirus with respect to disease severity in infants [14]. Nearly all humans are exposed to astrovirus by the age of five [15–17], and recent wastewater data indicate that astrovirus is shed at high levels [18].

Previous histological analyses of murine and turkey models have shown no signs of cell death following astrovirus infection [19–22]. Moreover, in vitro studies of human astrovirus (HAstV) have demonstrated a lack of cell death or lysis when viral particles are released [23,24]. Rather, a more recent study has observed that virus-associated large extracellular vesicles are secreted following HAstV infection [25]. These observations suggest an uncharacterized non-lytic pathway of virus release. A unique feature of astroviruses is their ability to infect goblet cells—specialized epithelial cells that secrete mucus--shown in human enteroids and mouse models [26–29]. Notably, peak murine astrovirus (MuAstV) infection coincided with increased mucus secretion, which occurs at 7 days post-infection (dpi) [26]. These findings raise the possibility that astroviruses utilize the goblet cell secretory pathway for egress.

Goblet cells synthesize mucin 2 (MUC2), a heavily glycosylated protein that forms a protective barrier on top of the intestinal epithelium [30,31]. The folding of MUC2 relies on key ER chaperone proteins, such as anterior gradient 2 (AGR2), that when lost, leads to defects in mucin biosynthesis [32–34]. In preparation for release, MUC2 is packaged into mucin granules that fill the apical region of the cell, known as the theca, that give the cell its beautiful cup shape. Mucus secretion is a carefully regulated process, controlled by ER stress [35] and signal transduction from microbial stimuli [36] as well as secretagogues, which increase intracellular Ca^2+^ and trigger compound secretion characterized by rapid fusion of mucin granules [37]. While several pathogens have evolved strategies to contend with mucus [38–41], no viruses to date have been reported to use mucus secretion as a pathway for egress.

In the following study, we examined both in vivo and human cell culture models of astrovirus infection to determine whether mucus secretion and viral egress are coordinated processes and whether modulating mucus secretion would impact viral egress. Together, these results reveal a previously unrecognized egress strategy among non-enveloped enteric viruses and uncover a new facet of host-virus interactions in the gut.

## Results

### MuAstV egress correlates with goblet cell mucus secretion

In a previous study, we examined the cellular tropism of MuAstV using in situ hybridization (ISH) with a MuAstV genome-specific probe and identified goblet cells as a major target for infection in the small intestine of C57BL/6J mice [26]. In many of the microscopy images, we noted frequent instances of virus particles spewing out of goblet cells, much like mucus (S1A Fig). Based on this observation, we next wanted to quantitatively assess whether mucus secretion and viral egress were co-occurring events. To maximize the number of infected goblet cells we could observe by microscopy, we performed our analysis with immunocompromised mice, which establish robust and persistent MuAstV infections and therefore, have reduced variability as compared to the kinetic phases of infection in C57BL/6J mice [20]. We began by examining muMT mice, which lack mature B cells, that were purchased from a commercial vendor and imported into our mouse facility. Upon arrival, we found that the mice were already infected with MuAstV based on screening of fresh feces by RT-qPCR. This was unsurprising given that MuAstVs are commonly detected in mice from commercial facilities [42]. We next used serial imaging with FITC-labeled wheat germ agglutinin (WGA), a lectin that binds to N-acetylglucosamine residues on mucin, to first identify goblet cells in the duodenums of muMT mice, followed by ISH to identify infected cells. Notably, the ISH probe that we previously designed to target ORF2 from MuAstV strains SJ001 and SJ002, used for experimental infections in C57BL/6J mice [20], also bound to the MuAstV strain detected in muMT mice, and this strain similarly showed a tropism for goblet cells. We next classified these infected cells based on whether they were secreting mucus, filled with mucus, or empty, according to the location of FITC-WGA signal and then classified based on whether virus was found inside the cell or egressing (Fig 1A). The majority of enumerated goblet cells were secreting mucus (92%) and among this group, almost all (95%) showed virus egressing (Fig 1B). The remaining 8% of cells were either filled with mucus or empty and only had virus inside. Interestingly, after we quantified the secretion status of the uninfected goblet cells in these images, we found significantly more infected cells secreting mucus and significantly fewer infected cells that were mucus-filled or empty (S1B Fig).

**Fig 1:**
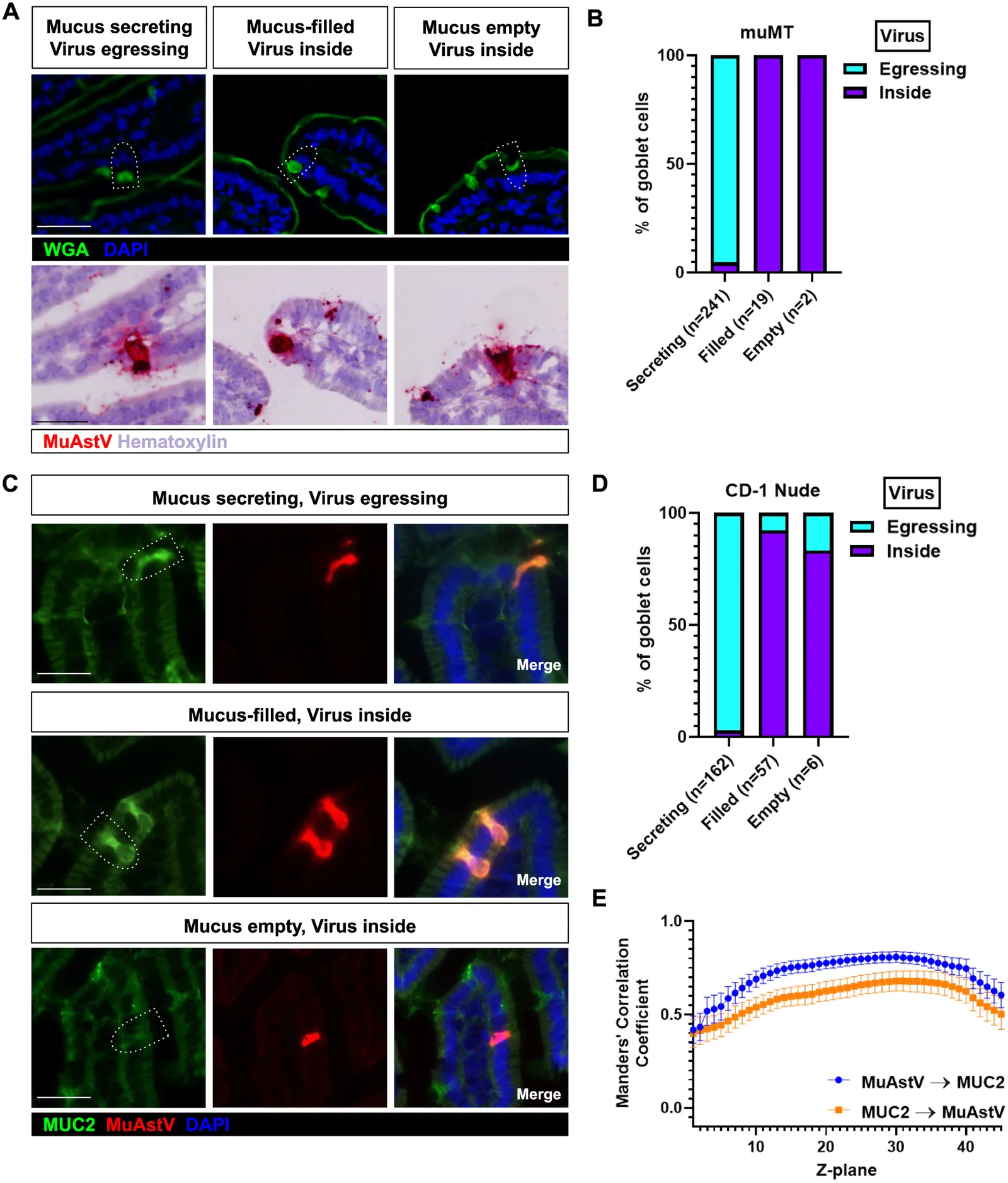
MuAstV egress correlates with mucus secretion in persistently infected immunocompromised mice. **A)** Serial imaging of duodenal tissues from muMT mice stained with WGA-FITC (green) and DAPI (blue) followed by ISH with a MuAstV genome-specific probe (red). Representative images show the three most common combinations of virus and mucus cell states, scale = 10 μm. **B)** Percent of infected goblet cells classified according to virus and mucus cell states (n = 5 muMT mice, n = 262 goblet cells). **C)** Representative images of ISH with a fluorescently labeled MuAstV genome-specific probe (red), MUC2 immunofluorescence (green), and DAPI (blue) collected from CD-1 Nude duodenal tissues, scale = 10 μm. **D)** Percent of infected goblet cells classified according to virus and mucus cell states (n = 5 CD-1 Nude mice, n = 225 goblet cells). **(E)** Manders’ correlation coefficients ± SEM calculated for overlapping signal between MuAstV and MUC2 across a z-stack centered around the middle of 22 infected secreting goblet cells imaged from CD-1 Nude mice.

We next examined CD-1 Nude mice, which lack a thymus and are T cell-deficient. Upon arrival, the mice tested negative for MuAstV, so we first inoculated them by oral gavage (strain SJ001 [20]) before analyzing the infected goblet cells within their duodenums once they became persistently infected. To avoid having to perform serial imaging, we switched to using a fluorescently labeled MuAstV-specific probe for ISH followed by immunostaining for MUC2 (Fig 1C). We performed the same mucus secretion and virus location analysis on the images collected by microscopy and again found that the majority (72%) of enumerated goblet cells were secreting mucus. Among this group of secreting cells, 97% had virus egressing (Fig 1D). The remaining mucus-filled (25%) or empty goblet cells (3%) predominantly had virus inside. In contrast to muMT mice, after we quantified the secretion status of the uninfected goblet cells in these images, we found no significant differences in the percentage of cells that were secreting mucus, mucus-filled, or empty (S1C Fig). We next used Manders’ correlation coefficient to measure the signal overlap between MUC2 and MuAstV within the infected cells quantified from CD-1 Nude mice. We observed strong correlations between MuAstV signal and MUC2, ranging from 0.45 to 0.85 (Fig 1E). There were slightly lower correlations between MUC2 with MuAstV (0.45 to 0.75), indicating that not all MUC2 within the cell colocalizes with virus. Overall, these findings indicate that viral egress and mucus secretion appear to be coordinated events within infected goblet cells.

### Goblet cell mucus secretion correlates with MuAstV infection kinetics

Unlike immunocompromised mouse strains, C57BL/6J mice do not establish persistent infections and therefore can serve as a model to understand how viral kinetics correlate with goblet cell secretion patterns after the acute phase of infection [20]. We previously observed that peak MuAstV infection in C57BL/6J mice at 7 days post-infection (dpi) correlated with increased mucus secretion [26]. We next questioned whether the increase in mucus secretion would be sustained throughout the infection of C57BL/6J mice or whether secretion levels would return to baseline during viral clearance based on the replication kinetics we previously examined [20]. To address these questions, we orally inoculated male and female C57BL/6J weanlings with MuAstV and then used MUC2 immunofluorescence to examine duodenal tissues collected at post-peak infection time points (10 and 18 dpi) as well as at viral clearance (28 dpi). We initially noted more mucus in the lumen of animals collected at 10 dpi relative to uninfected animals and animals at 18 and 28 dpi (Fig 2A). We next examined goblet cells based on their morphology and whether they appeared to be mucus-filled, secreting mucus, or empty (Fig 2B). We observed a significant increase in the percentage of empty goblet cells at 10 dpi (21%) and 28 dpi (9%), but not at 18 dpi (7%), as compared to uninfected controls (5%) (Fig 2C). These data indicate that goblet cells continue to exhibit increased mucus secretion right after peak infection and appear empty at 10 dpi, indicating a depletion of intracellular mucin stores, but this largely returns to baseline levels at later time points during viral clearance. Interestingly, we also found that *Muc2* expression significantly decreased at 10 dpi in epithelial cells isolated from the duodenums of infected mice as compared to uninfected controls, whereas there was no significant difference in *Muc2* expression at 18 and 28 dpi (Fig 2D). To rule out that the reduced *Muc2* expression we observed was not due to a loss of goblet cells at 10 dpi, we quantified the number of goblet cells per villus as well as the number of villi per field-view at these same timepoints and found no significant difference as compared to uninfected mice (Fig 2E-F). We also examined the expression of *Spdef*, a key transcription that promotes goblet cell differentiation [43], and found no significant change in expression throughout the timepoints (Fig 2G), indicating that MuAstV infection does not affect goblet cell frequencies or this phase of goblet cell differentiation. Overall, these findings collectively indicate that mucus secretion correlates with MuAstV infection kinetics.

**Fig 2:**
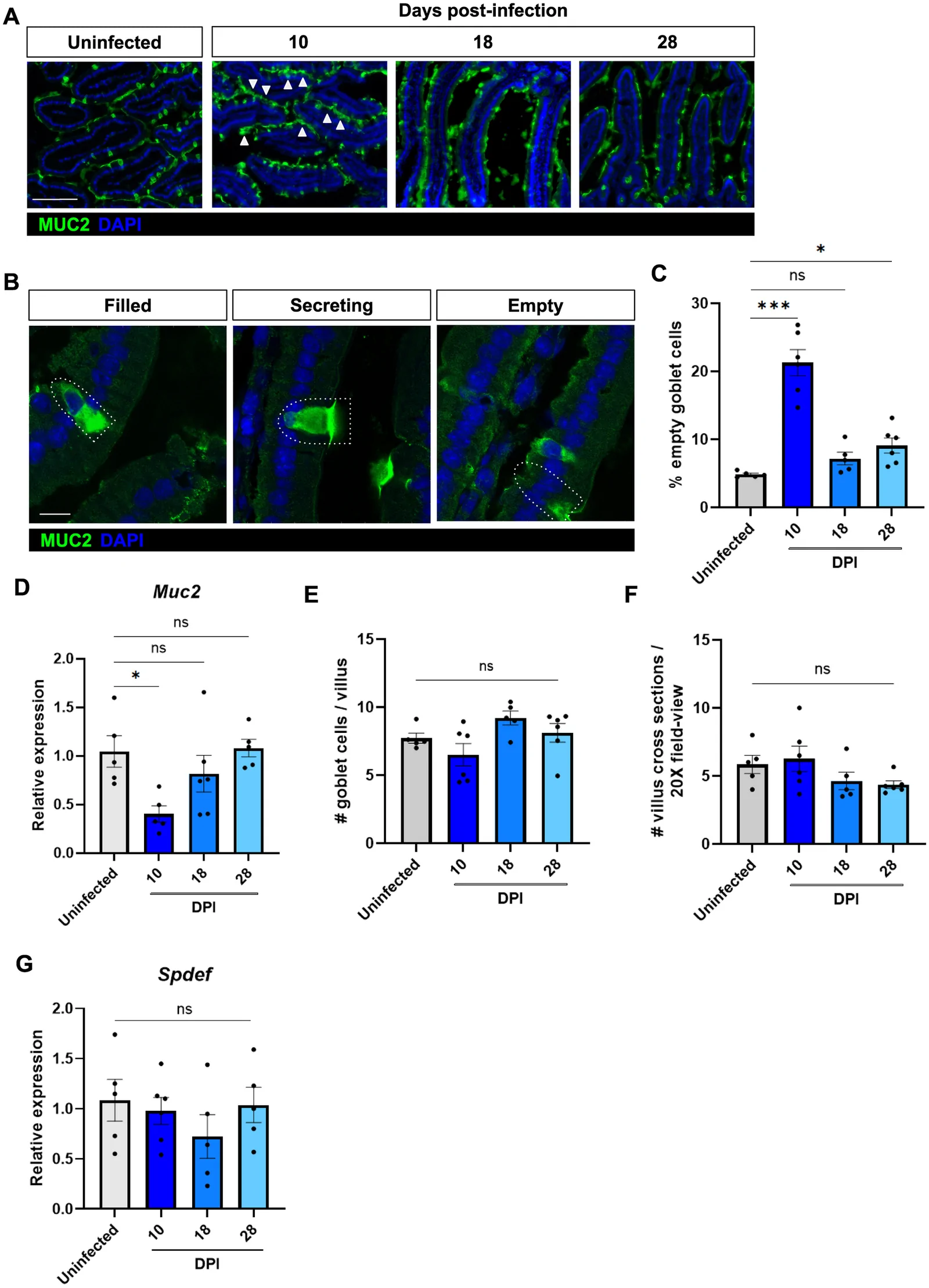
Goblet cell mucus secretion correlates with MuAstV infection kinetics. **(A)** Representative images of duodenal tissue immunostained for MUC2 (green) and counterstained with DAPI (blue) at all time points. Arrowheads indicate areas of mucus in between villi in the lumen. Scale bar = 100 μm. **(B)** Representative images of duodenal tissue section, highlighting an example of a filled, secreting and empty goblet cell immunostained for MUC2 (green) and DAPI (blue), scale bar = 10 μm. **(C)** Percent of goblet cells that were classified as empty in duodenal tissue sections from uninfected and infected animals (n = 5-6 mice/group). **(D)** *Muc2* expression in duodenal epithelial cells at time points normalized to *Hprt* and relative to uninfected animals (n = 5-6 mice/group). **(E)** Number of goblet cells per villus in uninfected and infected duodenal tissue sections (n = 5-6 mice/group). **(F)** Number of villi per 20X field-view image in uninfected and infected duodenal tissue sections (n = 5-6 mice/group). **(G)** *Spdef* expression in duodenal epithelial cells at time points normalized to *Hprt* and relative to uninfected (n = 5-6 mice/group). All data are presented as mean ± SEM. Statistical testing for **(C)** Welch’s ANOVA and (D-E & G) one-way ANOVA or **(F)** Kruskal-Wallis test with Dunnett’s multiple comparisons tests, ***p < 0.001, *p < 0.05; ns, non-significant.

### Loss of *Agr2* decreases MuAstV shedding and infection

Given the correlation we observed between mucus secretion and viral egress, we next questioned whether reducing mucus secretion would result in reduced virus shedding and local spread of the infection. To test this, we used mice lacking anterior gradient 2 (AGR2), a key ER chaperone that is critical for mucin biosynthesis [32–34]. We first evaluated the anticipated phenotype of this mouse model through histological analysis. Fluorescence microscopy revealed punctate MUC2 staining in goblet cells from *Agr2*^*-/-*^ animals, in contrast to the mucus-filled goblet cells in *Agr2*^*+/+*^ littermates (Fig 3A). This mirrored previous findings that showed abnormal goblet cell morphology that was consistent with disrupted mucin biosynthesis in *Agr2*^*-/-*^ animals [33]. We additionally measured *Muc2* expression from isolated duodenal epithelial cells and found that transcript levels were significantly reduced in *Agr2*^*-/-*^ mice compared to *Agr2*^*+/+*^ littermates (Fig 3B), as was previously described [32]. This is also consistent with a recent study that showed reduced *Agr2* expression following the loss of *Xbp1* in goblet cells triggered IRE1 endonuclease activity, which is normally inhibited by AGR2, resulting in *Muc2* mRNA decay [44]. However, to rule out the possibility that reduced *Muc2* expression was not due to a loss of goblet cells in *Agr2*^*-/-*^ mice, we quantified goblet cell numbers per villus by immunofluorescence microscopy and, surprisingly, found a significant increase in the number of MUC2 + cells in the *Agr2*^*-/-*^ mice relative to *Agr2*^*+/+*^ littermates (Fig 3C). Although goblet cell hyperplasia has not been reported previously in *Agr2*^*-/-*^ mice, these findings could be indicative of Paneth cell hyperplasia, which was previously noted in this mouse model [33]. We also examined *Spdef* expression and found no significant change in expression in the *Agr2*^*-/-*^ mice relative to *Agr2*^*+/+*^ littermates (Fig 3D). Overall, these data indicate that *Agr2*^*-/-*^ mice are a suitable model for examining how disrupted mucin biosynthesis affects MuAstV replication.

**Fig 3:**
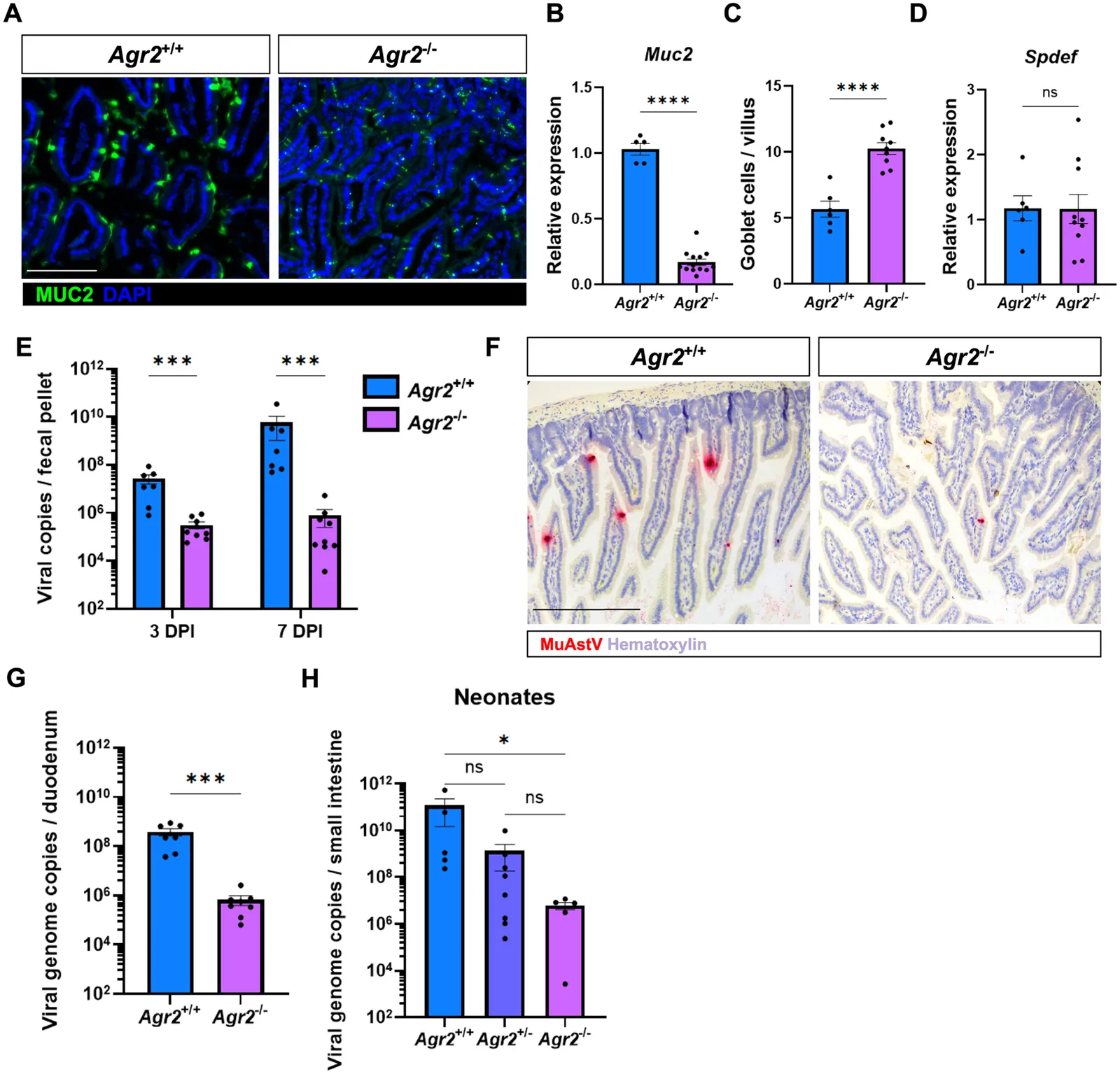
Loss of *Agr2* and decreased mucus secretion lead to decreased MuAstV infection. **(A)** Representative images of duodenal tissue from *Agr2*^*+/+*^ and *Agr2*^*-/-*^ littermates immunostained for MUC2 (green) and counterstained with DAPI (blue). Scale bar = 100 μm (n = 5-6 mice/group). **(B)** *Muc2* expression normalized to *Hprt* in duodenal epithelial cells isolated from *Agr2*^*-/-*^ relative to *Agr2*^*+/+*^ littermates (n = 5-9 mice/group). **(C)** Goblet cells enumerated per villus from *Agr2*^*+/+*^ and *Agr2*^*-/-*^ duodenal tissues (n = 6-9 mice/group, n = 60 villi/animal). **(D)** Fold change in *Spdef* expression relative to *Hprt* in duodenal epithelial cells isolated from *Agr2*^*+/+*^ and *Agr2*^*-/-*^ littermates (n = 6-10 mice/group). **(E)** Viral genome copies per fecal pellet collected from *Agr2*^*+/+*^ and *Agr2*^*-/-*^ littermates at 3 and 7 dpi (n = 7-8 mice/group). **(F)** Representative images of MuAstV genome-specific ISH (red) on duodenal tissue from *Agr2*^*+/+*^ and *Agr2*^*-/-*^ littermates at 7 dpi. Scale bar = 50 μm (n = 5 mice/group). **(G)** Viral genome copies per duodenum collected from *Agr2*^*+/+*^ and *Agr2*^*-/-*^ littermates at 7 dpi (n = 7-8 mice/group). **(H)** Viral genome copies per small intestine collected from PND 14 *Agr2*^*+/+*^, *Agr2*^*+/-*^, and *Agr2*^*-/-*^ littermates at 7 dpi (n = 5-8 mice per group). All data are presented as mean ± SEM. Statistical testing for **(B-D)** Welch’s t-test, **(E)** Mann-Whitney U tests with Holm-Šidák method for multiple comparisons, **(G)** Mann-Whitney U test, and **(H)** Kruskal-Wallis test; ****p < 0.0001, ***p < 0.001, *p < 0.05; ns, non-significant.

We next inoculated male and female *Agr2*^*-/-*^ and *Agr2*^*+/+*^ weanlings with MuAstV and monitored virus shedding in feces collected at 3 and 7 dpi. We found there was a significant reduction in MuAstV shed in the *Agr2*^*-/-*^ mice relative to *Agr2*^*+/+*^ littermates at both timepoints (Fig 3E). Unlike in the *Agr2*^*+/+*^ mice, there was no increase in shedding from 3 to 7 dpi (Fig 3E). Given this finding, we next questioned whether reduced virus shedding would result in decreased spread within the tissue. To address this question, we performed MuAstV-specific ISH and observed very few infected cells in the *Agr2*^*-/-*^ mice as compared to *Agr2*^*+/+*^ littermates at 7 dpi (Fig 3F). Consistent with minimal ISH signal, we also found that there was a significant reduction in MuAstV detected in the duodenal tissue of *Agr2*^*-/-*^ mice relative to *Agr2*^*+/+*^ littermates at this same time point (Fig 3G). We additionally examined MuAstV infection in postnatal day (PND) 7 *Agr2*^*-/-*^, *Agr2*^*+/-*^, and *Agr2*^*+/+*^ littermates. Similar to what we observed in weanlings, there was significantly less virus in the small intestines from *Agr2*^*-/-*^ pups relative to *Agr2*^*+/+*^ littermates at 7 dpi (corresponding to PND 14) (Fig 3H). Virus levels in heterozygotes were also slightly lower than *Agr2*^*+/+*^ mice, but this was not statistically significant (Fig 3H). Overall, these data indicate that MuAstV was able to enter goblet cells lacking AGR2 but had a reduced capacity to shed and propagate the infection within the small intestine.

### Mucus secretion triggered by carbachol increases MuAstV viral egress

We next examined whether stimulating mucus secretion would also trigger MuAstV release. To test this, we used carbachol (Cch), which is a structural analog of acetylcholine and is a well-characterized secretagogue [45]. To first establish the dose of Cch to use in our weanling model, we delivered 125 μg/kg Cch or vehicle control (PBS) via intraperitoneal injection into C57BL6/J animals and then collected duodenal tissue 45 minutes later to quantify the percentage of secreting goblet cells by histology (Fig 4A). We found that the percentage of secreting or empty goblet cells increased by 23% in Cch-treated mice relative to vehicle-treated mice (Fig 4B), indicating that the dose we used was sufficient to stimulate mucus secretion. We next inoculated male and female weanling C57BL6/J mice with MuAstV and at 7 dpi, we treated them with Cch or vehicle before harvesting their small intestinal contents to quantify virus released into the lumen (Fig 4C). We found that there was an average 5.8-fold increase in the amount of MuAstV in the intestinal contents following Cch treatment compared to vehicle, although this was not statistically significant (p = 0.0522, Fig 4D). We observed considerable variability in the effect of Cch on virus release, with 8 out of 20 Cch-treated mice exhibiting no increase as compared to vehicle-treated mice. This could in part be explained by the variable levels of infection across the experimental and control animals. In addition, this could be due to Cch exerting a lesser effect on goblet cells within the villi, which are the primary target for MuAstV [46], as compared to the crypt [47]. Alternatively, it is also possible that the addition of Cch may have a lesser effect at this time point (7 dpi), when mucus secretion is already heightened above baseline [26]. Even so, these results suggest a trend whereby stimulating mucus secretion can increase MuAstV release.

**Fig 4:**
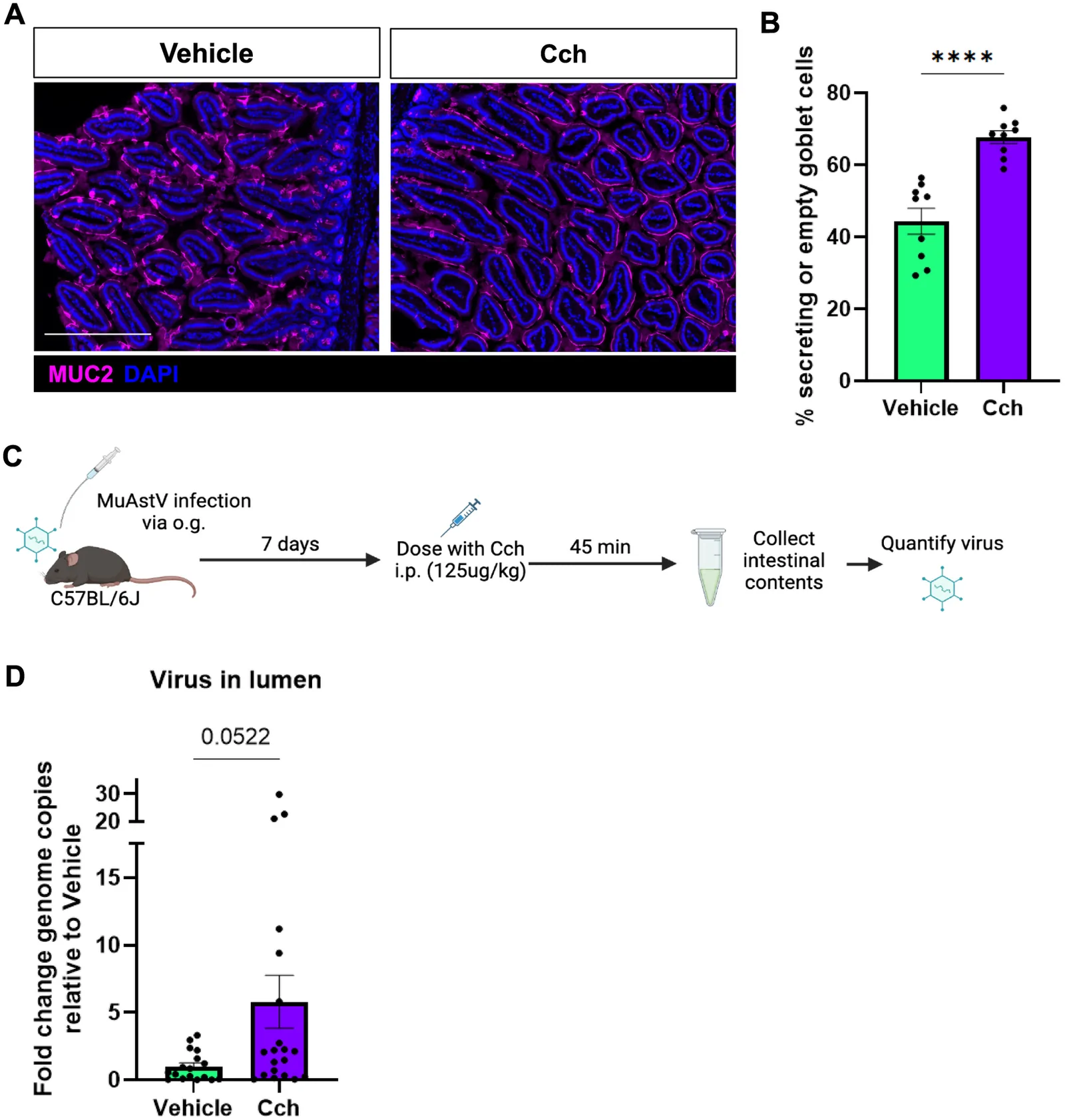
Carbachol induces mucus and MuAstV egress. **A)** Representative images of duodenum tissue from vehicle (PBS) and carbachol (Cch)-treated mice immunostained for MUC2 (magenta) and counterstained with DAPI (blue). Scale bar = 100 μm. **(B)** Percentage of mucus-secreting or empty goblet cells in vehicle and Cch-treated mice quantified from images as shown in panel A (n = 9 mice/group, ~ 160 villi analyzed/mouse). **(C)** Schematic of MuAstV infection and quantifying the effects of Cch treatment on virus levels released into the lumen. Created with BioRender. **(D)** Viral genome copies per 500 μL of small intestinal contents collected from vehicle and Cch-treated mice (n = 19-20mice/group) at 7 dpi. All data are presented as mean ± SEM. Statistical testing for **(B)** Welch’s t-test and **(D)** Mann-Whitney U test; ****p < 0.0001.

### Human goblet-like cell lines are susceptible to HAstV1 infection

Caco-2 cells, a heterogenous enterocyte-like colorectal cancer cell line, have served as the primary in vitro model for studying human astrovirus replication [48]. However, recent work has indicated that goblet cells are also susceptible to human astrovirus serotype 1 (HAstV1) infection in an enteroid model [29]. We next questioned whether HAstV1 could use a similar pathway for egress as MuAstV, but addressing this question required us to first establish a suitable in vitro model. To this end, we tested whether the human mucus-producing colorectal cancer cell lines, HT29-MTX-E12 and LS174T, were susceptible to HAstV1 infection, as neither cell line had been previously evaluated [49]. These cell lines serve as suitable in vitro models of goblet cell function, as they maintain the ability to synthesize, package, and secrete mucins in a manner that reflects goblet cell physiology [50]. We initially examined the goblet cell phenotype of these cell alines in comparison to Caco-2 cells and found that both HT29-MTX-E12 and LS174T cells expressed significantly higher levels of *MUC2* transcript (Fig 5A). We also examined the presence of MUC2 by immunofluorescence microscopy using two antibodies that target the C-term and PTS domains, respectively. Caco-2, HT29-MTX-E12, and LS174T all showed positive staining for the MUC2 C-term, which shares homology with other mucins, but only LS174T showed positive staining for the MUC2 PTS domain, which is distinctive in comparison to other mucins (Fig 5B). These findings demonstrate that HT29-MTX-E12 and LS174T cells both exhibit a more goblet cell phenotype than Caco-2 cells.

**Fig 5:**
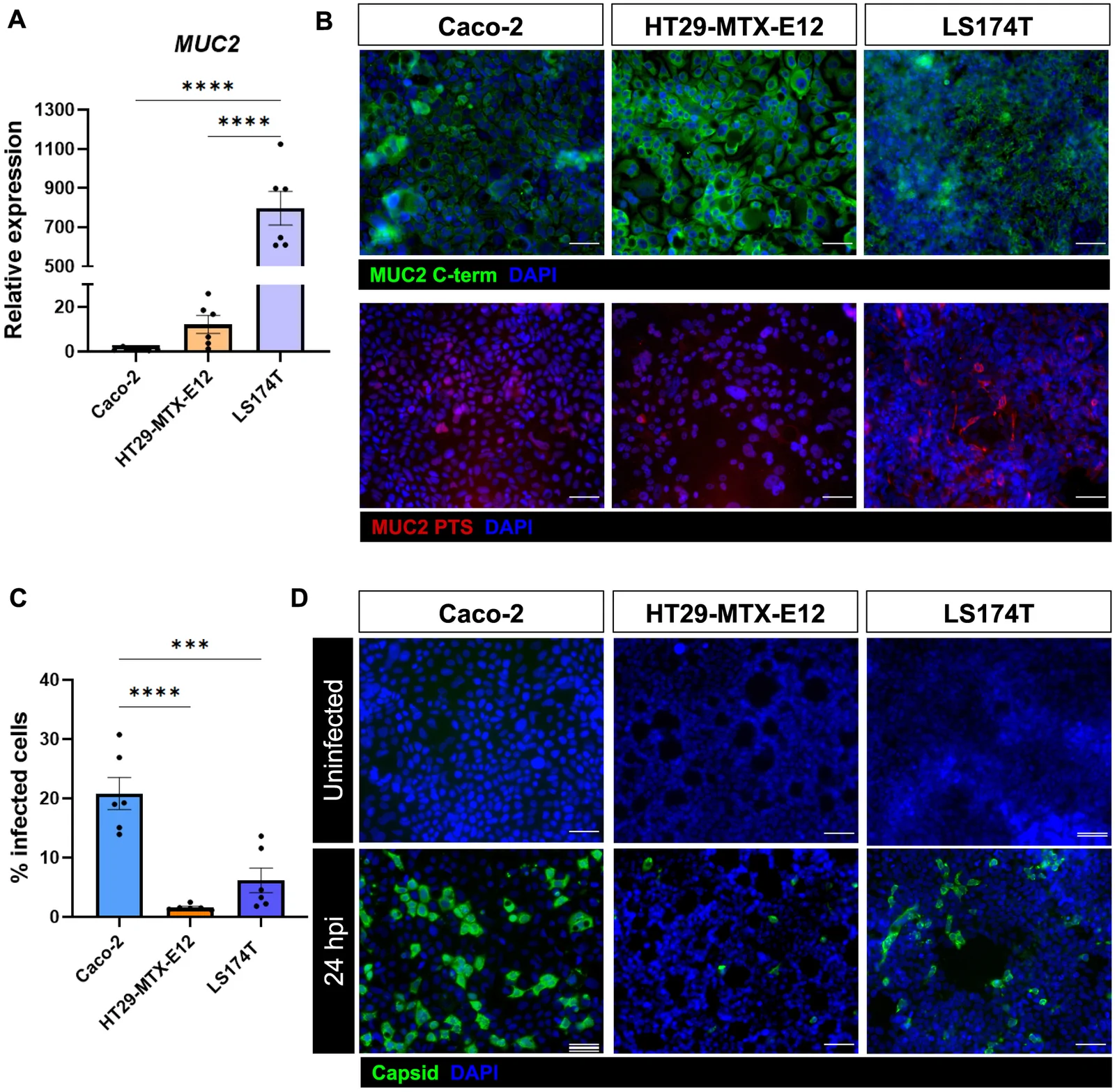
Human goblet-like cell lines are susceptible to HAstV1 infection. **(A)** *MUC2* expression normalized to *GAPDH* in HT29-MTX-E12 and LS174T cells relative to Caco-2 cells. **(B)** Representative immunofluorescence microscopy images of MUC2 C-term (green) or MUC2 PTS domain (red) with DAPI counterstain (blue). Scale bar = 100 µm. **C)** Cells infected with HAstV1 and imaged 24 hours post-infection (hpi) for HAstV1 capsid protein (green) and DAPI (blue). Representative images of the infection are shown in **(D)**. Scale bar = 100 µm. All data are presented as mean ± SEM. Statistical testing for (A) and (C) one-way ANOVA with Dunnett’s multiple comparisons test; ****p < 0.0001, ***p < 0.001.

We next infected each cell line with HAstV1 (MOI = 0.5) and quantified the percent infection at 24 hours post-infection (hpi), representing the end of a complete replication cycle [51], by immunofluorescence microscopy with an antibody targeting HAstV1 capsid (Fig 5C,D). We observed that HT29-MTX-E12 (1.6%) were the least infected relative to Caco-2 (20.8%), followed by LS174T cells (6.2%) (Fig 5C). Because Caco-2 and HT29-MTX-E12 can form monolayers, we additionally tested whether growing them at an air-liquid interface after a 1 and 2-week culture period would enable a model to examine HAstV1 infection and mucus secretion (S2A-L Fig). While we found that ALI conditions generally resulted in modest increases in *MUC2* expression (S2A, S2D, S2D, and S2J Fig), these conditions only enhanced the levels of virus in the HT29-MTX-E12 (S2C, S2F Fig), although the effect was variable and did not reach statistical significance. Interestingly, in the 2-week ALI cultures for HT29-MTX-E12 and Caco-2 cells (S2E, S2K Fig), we observed increased *MUC2* expression after the infection, but this was only statistically significant for Caco-2 cells. Overall, we identified that LS174T cells proved to be a suitable model for our subsequent experiments examining HAstV1 egress and mucus secretion.

### HAstV1 and MUC2 colocalize in LS174T cells and within mucin granules

We next used confocal microscopy to examine HAstV1 colocalization with MUC2 in the cell and within mucin granules. We infected LS174T cells (MOI = 1) and at 24 hpi, trypsinized them and allowed them to air-dry on coverslips in order to get an even monolayer before performing immunostaining and imaging (Fig 6A). We quantified colocalization of MUC2 and HAstV1 capsid and found that the signal overlap was high for both MUC2 relative to capsid and vice versa (75% and 77% overlap, Fig 6B). We next performed a mucin granule isolation [36] from infected and uninfected LS174T cells harvested at 24 hpi, to test whether there was detectable virus present with mucin granules. Based on co-staining for MUC2 and HAstV1 capsid, we observed particles that were consistent with the size of mucin granules (500–1500 nm) [52] and appeared to contain both MUC2 and virus, which was not observed in particles isolated from uninfected LS174T cells (Fig 6C) or uninfected MRC-5 cells, a human fibroblast cell line that does not express MUC2 (S3 Fig).

**Fig 6:**
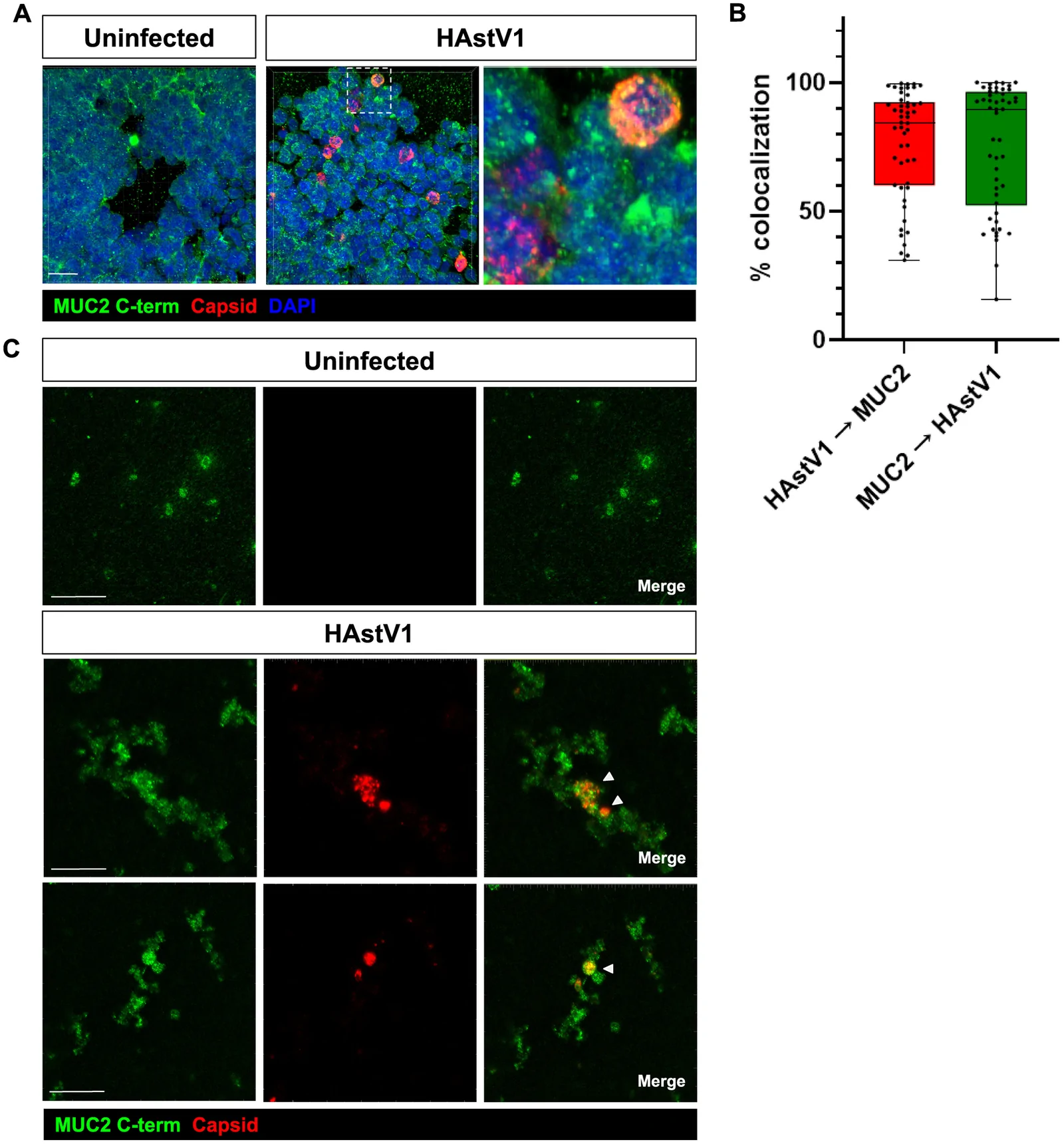
HAstV1 and MUC2 colocalize in LS174T cells and with mucin granules. **(A)** 3D confocal microscopy images of HAstV1-infected LS174T cells immunostained for MUC2 (green) and HAstV1 capsid (red) at 24 hpi. Scale bar = 10 µm. Inset shows a zoom-in on an infected cell. Scale bar = 2 µm. **(B)** Percent of fluorescence signal overlapping between MUC2 and HAstV1 capsid within infected LS174T cells (n = 50 infected cells analyzed). **(C)** Mucin granules isolated from infected and uninfected LS174T cells immunostained for HAstV1 capsid (red) and MUC2 (green). Scale bar = 5 µm.

We next purified mucin granules from our crude isolations by sucrose gradient ultracentrifugation. We ran all fractions via western blot using anti-HAstV1 spike polyclonal rabbit sera and identified two fractions that contained VP70, the main intracellular form of capsid, in fractions 9 and 12 that potentially indicate granule-associated and free virus, respectively (Fig 7A). Because MUC2 is highly glycosylated and does not resolve in a clear band by SDS-PAGE, we used Fc gamma binding protein (FCGBP) as a mucin granule marker to examine whether the capsid-positive fractions also contained FCGBP. We initially tested the FCGBP antibody specificity via immunostaining of LS174T cells visualized by confocal microscopy (S4 Fig) before using it for a western blot to examine fractions 9, 12, and neighboring fractions as controls (Fig 7B). We only observed FCGBP detected in fraction 9, which was consistent with immunofluorescence images of these fractions, where we only observed clear co-staining for MUC2 and HAstV1 capsid in fraction 9 (S5 Fig, Fig 7C). Together, these data support a model in which nascent virions colocalize with MUC2 within LS174T goblet cells prior to release and can be co-purified with mucin granules.

**Fig 7:**
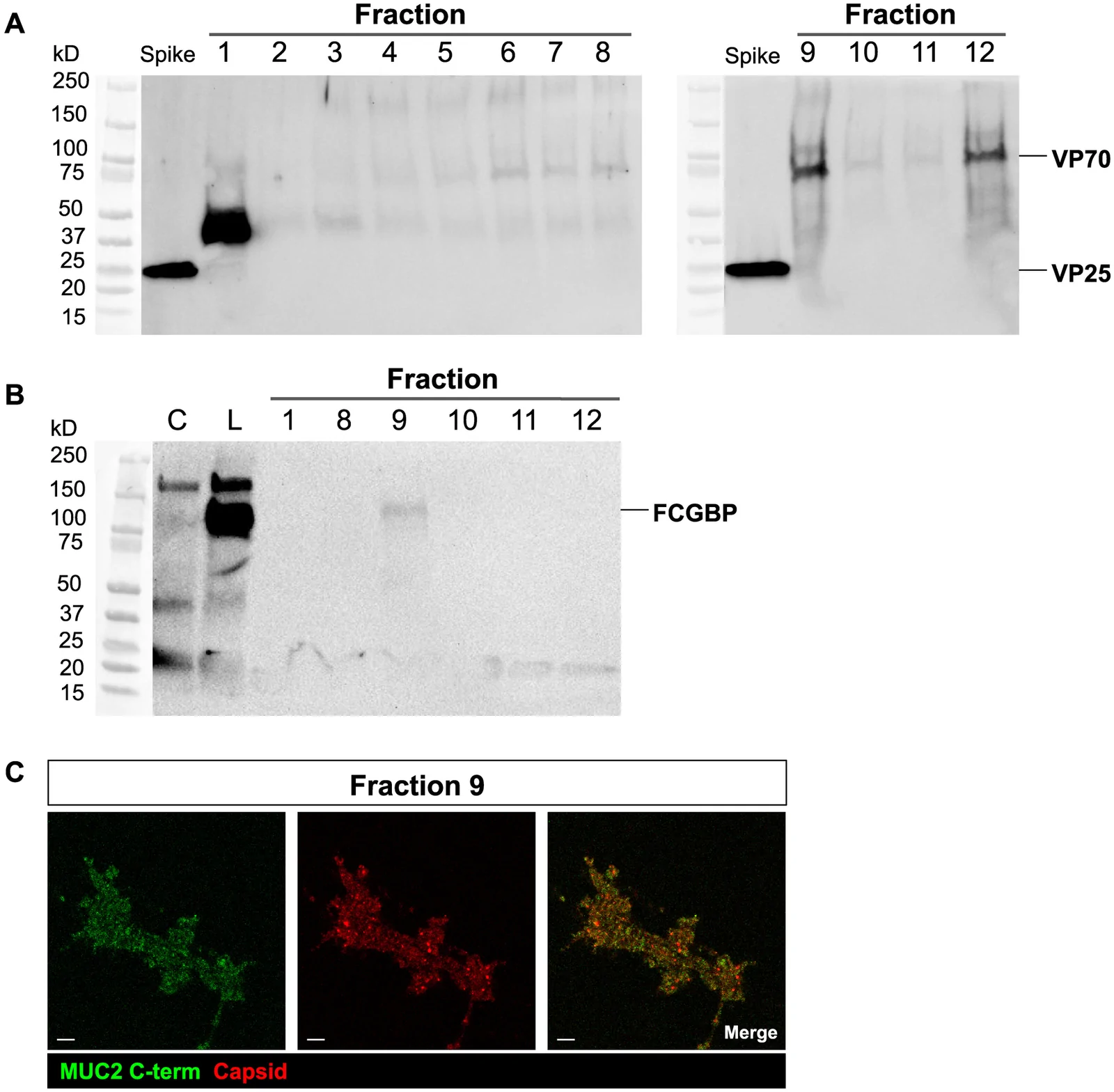
HAstV1 and mucin granules co-purify after density gradient ultracentrifugation. **(A)** Mucin granules isolated from HAstV1-infected LS174T cells, fractionated by sucrose gradient, and visualized by western blot probed with anti-HAstV1 rabbit hyperimmune sera. Expected sizes of viral capsid protein (VP) are indicated at right, with VP70 denoting intracellular processed capsid. Purified recombinant HAstV1 spike protein (VP25) serves as a positive control. **(B)** Western blot of selected purified mucin granule fractions from (A) that were removed from sucrose and probed for FCGBP. Expected size of FCGBP is indicated at right. Lysates from Caco-2 and LS175T cells serve as controls and are labeled as C and L, respectively. **(C)** Fraction 9 immunostained for MUC2 (green) and HAstV1 capsid (red) visualized by confocal microscopy. Scale bar = 10 µm.

### Stimulated mucus secretion enhances HAstV1 egress

We next examined whether Cch-stimulated mucus secretion could trigger HAstV1 release from LS174T cells. To ensure the Cch dose was sufficient to induce mucus secretion, we conducted a dot blot analysis using serial dilutions of supernatants collected from LS174T cells treated with 1) Cch, 2) pre-treatment with tropicamide, a muscarinic receptor antagonist, followed by Cch, or 3) vehicle (PBS) control alongside a dilution series of porcine gastric mucin that served as our positive control (Fig 8A). Following incubation of the membrane in alcian blue, which binds to acidic mucins, we observed a 1.22 increased intensity of blue stain in supernatants from cells treated with Cch as compared to vehicle-treated cells when loaded neat, which was close to the 1.74 increase observed with 200 µg/ml of porcine gastric mucin (Fig 8A). This effect from Cch was blocked by pre-treatment with tropicamide as indicated by reduced alcian blue intensity (0.81, Fig 8A). We next infected LS174T cells with HAstV1 (MOI = 0.5) and at 23 hpi, media was removed, and cells were treated with Cch, tropicamide, pre-treatment with tropicamide followed by Cch, or vehicle control. Cell supernatants collected after 1-hour treatment were titrated on Caco-2 cells to measure virus release. Treatment with Cch resulted in a significant increase in HAstV1 release from LS174T cells as compared to vehicle control (Fig 8B). Treatment with tropicamide resulted in no significant change in HAstV1 released into the supernatant relative to vehicle, but was able to block the effects of Cch, resulting in a significant decrease in HAstV1 titer compared to Cch alone (Fig 8B). We additionally tested the same Cch treatment with infected Caco-2 cells and noted an increase in virus titer, but this was not statistically significant compared to vehicle control (S3 Fig). Overall, these data mirror our findings in the MuAstV model, indicating that HAstV1 release can be triggered via stimulated mucus secretion.

**Fig 8:**
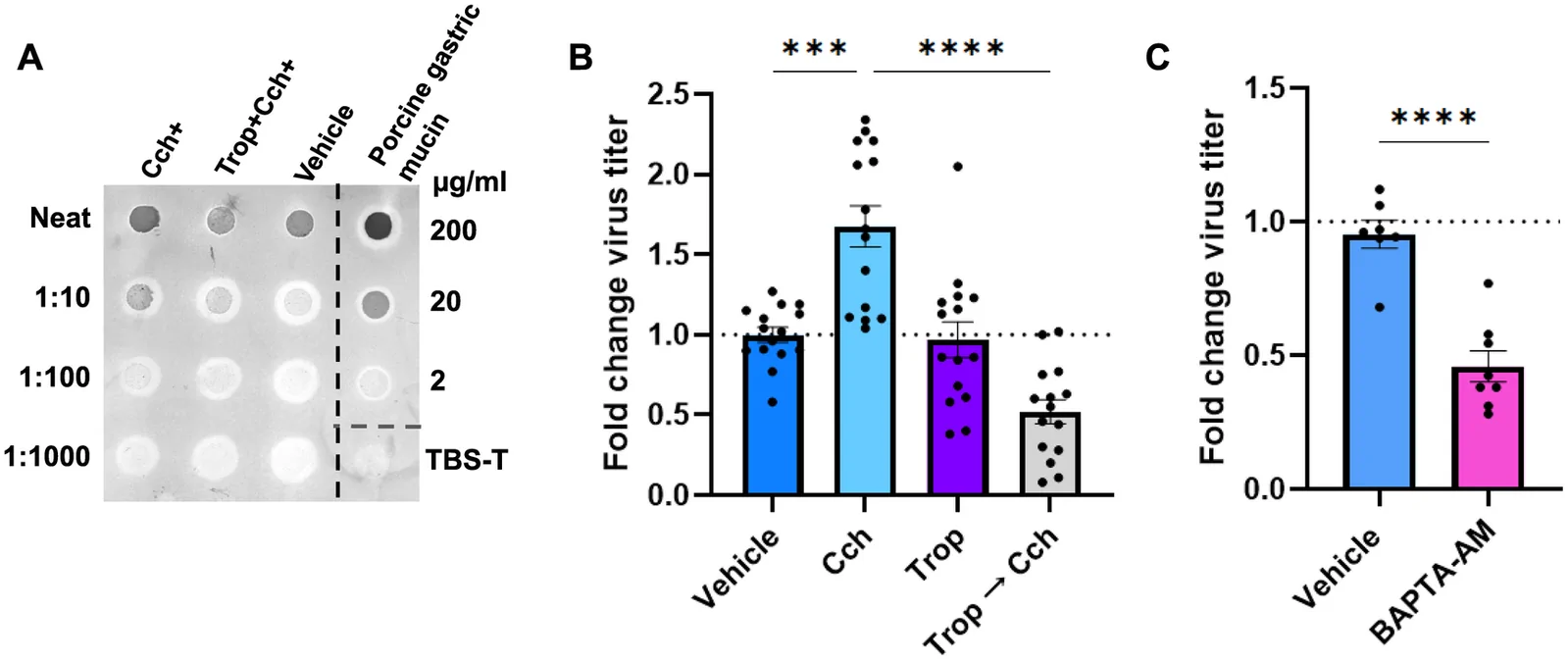
Stimulated mucus secretion enhances HAstV1 egress. **(A)** Dot blot with a dilution series of supernatants from LS174T cells treated with 1 mM Cch, 1 mM tropicamide + 1 mM Cch, or vehicle (PBS). Serial dilutions of porcine gastric mucin are shown as a positive control and TBS-T as a negative control. Blot was stained with alcian blue and imaged in greyscale. **(B)** Fold change in HAstV1 titer quantified from supernatants collected after a 1-hour incubation with 10 μM Cch, 1 mM tropicamide (Trop), or 1 mM Trop and 10 μM Cch from infected LS174T cells at 24 hpi relative to vehicle-treated cells. **(C)** Fold change in HAstV1 titer quantified from supernatants collected after a 30-minute incubation with 10 μM BAPTA-AM from infected LS174T cells at 24 hpi relative to vehicle (HBSS without Ca^2+^ Mg^2^)-treated cells. All data are presented as mean ± SEM. Statistical testing for **(B)** Welch’s ANOVA with Dunnett’s multiple comparisons test, **(C)** Welch’s t-test; ****p < 0.0001, ***p < 0.001.

Stimulated secretion is a Ca^2+^-dependent process that leads to rapid fusion and release of mucin granules [53]. We questioned whether intracellular Ca^2+^ release preceded HAstV1 egress, which would indicate that the virus could serve as a direct stimulus for secretion. To address this, we tested whether chelating intracellular calcium could inhibit virus release from LS174T cells. We infected cells with HAstV1 (MOI = 0.5) and at 23 hpi, media was removed and cells were treated with BAPTA-AM or vehicle control (HBSS without Ca^2+^ Mg^2+^) for 30 minutes. Cell supernatants were then titrated on Caco-2 cells to measure virus release. Treatment with BAPTA-AM significantly reduced the amount of HAstV1 in supernatants collected from LS174T cells as compared to vehicle (Fig 8C), indicating that human astrovirus release is Ca^2+^-dependent. Overall, these experiments indicate that viral egress is promoted by stimulated secretion and intracellular calcium signaling.

## Discussion

Astroviruses are a leading cause of pediatric diarrhea worldwide [14]. Despite their high prevalence, they are understudied, and many steps of their replication cycle remain uncharacterized, including their pathway(s) for egress. Here, we identified a novel, non-lytic process by which nascent astrovirus particles are released from goblet cells via mucus secretion. Critically, utilizing in vivo studies and a new in vitro model, we showed that both murine and human astroviruses are capable of exiting goblet cells using this pathway. Together, these findings highlight a new facet of enteric virus-host biology that allows for the efficient release of virus particles without triggering cell death. The interaction between astrovirus and goblet cell secretion appears distinct from other major enteric viruses. For instance, rotavirus infection is characterized by a significant reduction in the goblet cell population but little change in *Muc2* expression [54]. In contrast, our findings indicate that astrovirus promotes secretory activity to facilitate its own egress.

In this study, we observed that mucus secretion and viral egress were coordinated processes within infected goblet cells. Stimulated mucus secretion can be mediated by cholinergic agonists, as demonstrated here with carbachol, as well as other secretagogues and stimuli in the gastrointestinal tract [53]. Thus, it is possible that these different stimuli could aid astrovirus egress during an infection. However, it is also quite likely that increased mucus secretion following astrovirus infection is virus driven. While additional studies are needed to fully address this open question, we note here that in experiments where we chelated intracellular calcium, we observed less HAstV1 released from LS174T cells. It is also notable that we observed HAstV1 particles associated with purified mucin granules. This could support a model by which a viral protein enables intracellular calcium flux to trigger compound exocytosis and simultaneous release of virus with mucin granules. Future subcellular analyses are warranted to investigate whether astrovirus assembly runs parallel to or intersects with the formation of secretory granules in goblet cells.

The identification of a mucus-dependent viral egress pathway also has several broader implications for mechanisms that enable evasion of host immunity and/or enhance viral spread and transmission [55]. For example, it is possible that “hiding” amongst mucus enables a virus to evade host detection or has the potential to enhance entry into new cells, perhaps analogous to how rotavirus and norovirus utilize vesicle-cloaked virus clusters for entry [2]. Indeed, it has been previously shown that mucin enhances the thermostability of human astrovirus [56], which could aid the spread within and/or between hosts. In this study, we also noted that *Agr2*-deficient mice had impaired mucus secretion and reduced MuAstV shedding as compared to wildtype littermates at 3 dpi, and this led to a suboptimal infection based on virus levels assessed in the duodenum at 7 dpi. This could indicate that the virus was unable to spread efficiently to neighboring cells and establish a robust infection due to the lack of mucus. Additional studies are needed to illuminate these dynamic interactions.

This study should be framed in the context of a number of limitations. First, our *in vitro* experiments relied on mucin-producing colorectal cancer cell lines. While these cells are an established model for studying MUC2 biosynthesis and secretion, they do not fully represent the heterogeneity and complexity of goblet cells *in vivo*. Future studies with human and murine enteroids may provide additional insights into how the physical properties of the overlying mucus barrier influence viral spread. Second, while we demonstrate a role for calcium-dependent secretion in astrovirus egress, the specific viral proteins that facilitate the virus exiting with mucin granules remain to be determined. Finally, it is unclear how infected goblet cells sustain high production of both mucus and virus, and it remains an open question as to whether astrovirus infection drives the cell into ER stress response pathways. The consequences of such a cellular response could result in a disrupted mucus barrier, leading to increased susceptibility to bacterial and parasitic pathogens [57–61]. Thus, we question whether this contributes to astrovirus pathogenesis and is an important variable in co-infections [62], which were found to be highly prevalent in a multi-site birth cohort [14]. Overall, this work not only provides insights into the astrovirus replication cycle but also broadens our understanding of enteric viral pathogenesis, revealing opportunities to develop future antimicrobial therapies.

## Methods

### Ethics

All animal experiments were approved by the University of California, Santa Cruz Vivarium and Institutional Animal Care and Use Committee (protocol Cortv2406dn). The University of California Santa Cruz is fully accredited by the Association for the Assessment and Accreditation of Laboratory Animal Care International (AAALAC-I). It has an approved Animal Welfare Assurance Statement on file with the Office of Laboratory Animal Welfare (D16-00493). These guidelines were established by the Institute of Laboratory Animal Resources and were approved by the Governing Board of the U.S. National Research Council.

### Animals

Male and female C57BL/6J strain #000664 and *Agr2* KO mice strain #025630 were purchased from Jackson Laboratory and bred in-house. Female 4-week-old muMT strain #002288 and male 3-week-old CD-1 Nude mice strain #087 were purchased from Jackson Laboratory and Charles River Laboratories, respectively. All mice were housed in specific pathogen-free conditions (n = 2–5 mice/cage). For all experiments, before inoculation on day 0, mice were confirmed to be negative for MuAstV by RT-qPCR screening of fresh feces using the primer and probe system described below. The one exception was with muMT mice, which tested positive for MuAstV upon arrival from Jackson Laboratory. While the MuAstV virus from muMT mice was not sequenced, it was detected by the same primer/probe assay and ISH probe that was used to detect strain SJ001, which was used in the experimental infections of C57BL/6J mice, indicating that these viruses have shared homology. For all experiments, mice were co-housed according to genotype before experimentation.

### MuAstV infection and virus quantification

For experimental MuAstV infections, mice aged 3–4 weeks were orally inoculated with 100 µL of 0.22 μm filtered fecal filtrate (100 mg/mL), corresponding to 3.05 × 10^6 genome copies of MuAstV strain SJ001 (GenBank accession #MK395165). Fecal samples were collected with 2.0 mm zirconium oxide beads and stored at -80°C until RNA extraction. To quantify virus levels, feces and duodenum tissue samples were homogenized with 500 uL of PBS or 800 µL of Trizol using the Bullet Blender, and the clarified supernatant was collected following centrifugation at 18,000 x g for 1.5 min. RNA was extracted with the QIAmp Viral RNA Mini Kit (Qiagen) or the Directzol RNA Miniprep Kit (Zymo Research). MuAstV genome copies were quantified using TaqMan Fast Virus 1-step Master Mix for qPCR (Applied Biosystems) with primers (F: TACATCGAGCGGGTGGTCGC, R: GTGTCACTAACGCGCACCTTTTCA) and probe [(6-FAM)- TTTGGCATGTGGGTTAA- (MBGNFQ)] under the following conditions: 50°C for 5 min and 95°C for 20 s, followed by 40 cycles of 95°C for 3 s and 60°C for 30 s on a BioRad CFX Connect Real-Time System and compared to a g-block standard (Integrated DNA Technologies).

### Histological analyses

Duodenal samples were collected and placed in 10% formalin or Carnoy’s fixative (60% methanol, 30% glacial acetic acid, 10% chloroform) overnight before transfer to 30% sucrose and embedded in O.C.T. Tissues were sectioned at 6 µm thickness and probed using RNAScope 2.5 Chromogenic Assay and the RNAscope Multiplex Fluorescent Reagent Kit v2 with a MuAstV-specific probe according to the ACD Bio guidelines.

To analyze goblet cells and mucus secretion, tissue sections were also blocked with 0.5% bovine serum albumin (BSA) before immunostaining with anti-MUC2 polyclonal antibody C3 (1:100, GeneTex) followed by goat anti-rabbit Alexa Fluor 488 (1:1000, Invitrogen) or WGA-FITC (1:800, Vector Labs), and DAPI (Cell Signaling) prior to mounting with ProLong Diamond Antifade Mountant (Invitrogen). Slides were visualized with an Echo Revolve, Leica Widefield, Nikon NSPARC confocal, and/or Zeiss 880 confocal microscopes.

### Epithelial cell isolation and quantification of gene expression

Mouse duodenums were dissected and placed in DPBS on ice. The tissue was opened longitudinally, and the contents were washed out before the tissue was minced and added to strip buffer (10% FBS, 15 mM HEPES, 5 mM EDTA, 5 mM DTT in HBSS without Ca^2+^ Mg^2+^). Samples were shaken at 200 rpm for 20 min at 37°C, followed by vortexing at max speed. The samples were then strained through a 70 μm filter and washed with 10 mL of HBSS. Cells were spun down at 400 x g for 10 min and pellets resuspended in RLT lysis buffer before RNA extraction with the RNeasy Plus Mini Kit (Qiagen). PrimeTime qPCR primers (Integrated DNA Technologies) were used for murine *Spdef (*Mm.PT.58.31043304), *Muc2 (*Mm.PT.58.29496069.g*),* and *Hprt* (Mm.PT.58.32092191) transcript quantification on the BioRad CFX Connect Real-Time System.

### HAstV1 infection and virus quantification

Caco-2 cells were grown in MEM, 10% FBS, 2 mM L-glutamine, 1 mM sodium pyruvate, and 100 U/mL Penicillin-Streptomycin. HT29-MTX-E12 cells were grown in DMEM, 10% FBS, 2 mM L-glutamine, 1X non-essential amino acids, and 100 U/mL penicillin-streptomycin. LS174T cells were grown in EMEM, 10% FBS, and 100 U/mL penicillin-streptomycin. Prior to infections, Caco-2 cells were seeded in 96-well plates and grown for 7 days at 37°C and 5% CO_2_ to allow for differentiation, with a media change after 3–4 days. HT29-MTX-E12 and LS174T cells were seeded in 96-well plates and grown for 2–3 days at 37°C and 5% CO_2_ prior to infections. HAstV1 infections proceeded in serum-free media for 60–90 minutes before the inoculum was removed and replaced with serum-free media for 18–24 hrs. Infections were quantified after the cells were fixed in ice-cold methanol for 20 min at -20°C and then washed and stored in DPBS. Cells were blocked with 0.5% BSA for 30 min, then stained with the HAstV1 capsid-specific monoclonal antibody, 8E7 (1:10,000, Invitrogen), for 1 hour, followed by goat anti-mouse 488 (1:1000, Invitrogen) and DAPI (Cell Signaling). Cell supernatants were also collected at 18–24 hpi, and virus titers were quantified following activation with 10 μg/mL porcine trypsin (Sigma) for 30 min prior to absorption on Caco-2 cells. Infections and cell harvests proceeded as described above, with titers enumerated by a fluorescent focus unit assay as previously described [63]. Cells were visualized with an Echo Revolve microscope.

### Air-liquid interface cell culture and gene expression analysis

Caco-2 and HT29-MTX-E12 cells were seeded on transwell plates (Corning, 6.5 mm with 0.4 µm pore) for 7 or 14 days at 37°C and 5% CO_2_ to allow for differentiation with media changes every 3–4 days before being taken to air. Cells were kept at ALI for 7 days before HAstV1 infection (MOI = 0.5). Cells were harvested in RLT lysis buffer, and RNA was extracted with the RNeasy Plus Mini Kit (Qiagen). cDNA was generated with iScript cDNA Synthesis kit (Bio-Rad) and qPCR was conducted with QuantiTect primers (Qiagen) for human *MUC2* (QT01004675) and *GAPDH* (QT00079247). HAstV1 genome copies were quantified using TaqMan Fast Virus 1-step Master Mix for qPCR (Applied Biosystems) with primers (F: CCAGRCTCACAGAAGAGCAAC, R: CTTGCTAGCCATCRCACTTCTT) and probe [(6-FAM)- CATCGCATTTGGAGGGGAGGACC - (MBGNFQ)] under the following conditions: 50°C for 5 min and 95°C for 20 s, followed by 40 cycles of 95°C for 3 s and 60°C for 30 s on a BioRad CFX Connect Real-Time System and compared to a g-block standard (Integrated DNA Technologies).

### Carbachol and BAPTA-AM treatments

For experiments *in vivo*, mice were treated with 125 μg/kg carbachol (Thermo Scientific Chemicals) or vehicle (PBS) in a 100 μL intraperitoneal injection with a tuberculin syringe. Intestinal contents were collected from the entire length of the small intestine and stored with 2.0 mm zirconium oxide beads at -80°C prior to RNA extraction and virus quantification by RT-qPCR as described above. For experiments *in vitro*, cells were seeded in 96-well plates and infected with HAstV1 as described above. At 23 hpi, 1 mM tropicamide (Thermo Scientific Chemicals) was added to the treatment wells through a partial media exchange (50 μL was added and removed four times in a row to reduce disturbance to the monolayer). After a 15 min incubation, 10 µM carbachol was added and incubated for 30 min before supernatant was collected for virus quantifications. For experiments that used BAPTA-AM, cells were seeded in 96-well plates and infected with HAstV1 as described above. At 23 hpi, media was removed and replaced with 10 µM BAPTA-AM (Invitrogen) in HBSS (without Ca^2+^ Mg^2+^) or vehicle control (HBSS) for 30 min before supernatant was collected for virus quantifications.

### MUC2 immunostaining and colocalization analysis

Immunostaining was performed on LS174T, HT29-MTX-E12, and Caco-2 cells with anti-MUC2 PTS monoclonal antibody 966/1 (1:100, Invitrogen), which detects the middle repeat domain of MUC2 that is divergent from MUC5AC and MUC5B, for 1 hour, as well as anti-MUC2 polyclonal antibody C3 (1:100, GeneTex), which detects the C-terminus of MUC2, followed by goat anti-rabbit Alexa Fluor 488 (1:1000, Invitrogen) or goat anti-mouse Alexa Fluor 555 (1:1000, Invitrogen), respectively, for 1 hour, and mounted with ProLong Diamond Antifade Mountant (Invitrogen). For the colocalization analysis, LS174T cells were seeded in 24-well plates and grown to 80% confluency following two days in culture. Cells were infected with HAstV1 as described above and at 24 hpi, cells were trypsinized, spun down for 5 min at 300 x g and 100 µL was air-dried onto a glass slide for at least 3 hours or overnight before ice-cold methanol fixation and immunostaining. Slides were blocked with 0.5% BSA for 30 min and stained with anti-MUC2 polyclonal antibody C3 (1:100, GeneTex), HAstV1 capsid-specific monoclonal antibody, 8E7 (1:10,000, Invitrogen) for 1 hour, followed by goat anti-rabbit Alexa Fluor 488 (1:1000, Invitrogen), and goat anti-mouse Alexa Fluor 555 (1:1000, Invitrogen) for 1 hour, and mounted with ProLong Diamond Antifade Mountant (Invitrogen). Validation of the anti-FCGBP antibody (1:500, Cloud-Clone Corp) was performed by immunostaining LS174T cells grown on chambered TC-treated Ibidi coverslips. Cells were visualized with an Echo Revolve and/or Zeiss 880 confocal microscopes.

### Mucin granule isolation and analysis

As described by Gorman et al. [64], mucin granules were isolated from infected and uninfected LS174T cells. The human fibroblast cell line, MRC-5 [65], were also used as a negative control for the mucin granule isolation experiments. All cells were grown in five T-75 flasks and at 24 hpi, cells were trypsinized and pelleted by centrifugation for 5 min at 300 x g before resuspending in MES buffer (250 mM sucrose, 25 mM MES, 0.1 mM MgSO_4_, 2 mM EGTA, and 0.1 mM PMSF) and lysed with a Dounce homogenizer. The homogenate was then centrifuged at 300 x g for 5 min and the supernatant was collected, followed by a second spin at 300 x g for 10 min. The clarified supernatant was then centrifuged at 2,100 x g for 15 min and the crude mucin granules were resuspended in PBS and 100 µL was air-dried onto a glass slide for at least 3 hours or overnight before fixation and immunostaining as described above for MUC2 and HAstV1 capsid. For further purification, crude mucin granules were resuspended into 2 mL HEPES buffer (20 mM HEPES, 137 mM NaCl) and carefully placed on top of a sucrose gradient (2 mL of each 2 M sucrose, 1.5 M sucrose, 1 M sucrose, and 0.5 M sucrose). The samples were ultracentrifuged at 20,000 x g overnight in a Beckman Coulter Optima L-90K Ultracentrifuge with a SW41 Ti rotor. After centrifugation, 1 mL fractions were collected. The fractions were then prepared for western blot analysis by first denaturing in NuPAGE LDS Sample Buffer and Reducing Agent and then separated on a Bio-Rad Mini-PROTEAN TGX Stain-Free 4–15% Gel. Purified recombinant HAstV1 spike protein (VP25) (gift from Dr. Rebecca DuBois, University of California, Santa Cruz) was used as a positive control for detecting HAstV1 capsid and the fractions with clear positivity for capsid (fractions 9 and 12) as well as a few without robust signal (fractions 1, 8, 10 and 11) were washed three times in PBS (30 min spins at 37,000 x g) and prepared for western blot analysis probing for FCGBP along with Caco-2 and LS174T cell lysates as positive controls. Following SDS-PAGE, proteins were transferred to nitrocellulose membranes. Membranes were blocked with 2.5% BSA for 1 hour at room temperature and then incubated with HAstV1 spike rabbit anti-sera (1:500, kind gift from Dr. Rebecca DuBois, University of California, Santa Cruz) or blocked with 5% milk and then incubated with FCGBP rabbit polyclonal antibody (1:500, Cloud-Clone Corp) overnight at 4°C. Membranes were washed three times for 5 minutes each in TBS-T, then incubated with anti-rabbit IgG HRP (1:1,000, Cell Signaling) for 1 hour at room temperature followed by Pierce ECL Western Blotting Substrate for HAstV1 capsid or Pierce SuperSignal West Pico PLUS Chemiluminescent Substrate for FCGBP and imaged on a Bio-Rad ChemiDoc system. Purified granules were also analyzed by immunostaining for HAstV1 capsid and MUC2 as described above for crude granules. Slides were visualized with an Echo Revolve and/or Zeiss 880 confocal microscopes.

### Dot blot

LS174T cells were seeded in T-75 flasks and grown to 80% confluency over a period of two days. Cells were serum-starved for 1 hour at 37°C before treatment with 1 mM carbachol with or without 1 mM tropicamide, or PBS (vehicle control). Following a 1-hour incubation at 37°C, supernatants were collected and clarified at 500 x g for 5 min to remove cellular debris and then serially diluted before transferring to a nitrocellulose membrane with a Bio-Dot SF Microfiltration Apparatus (Bio-Rad). Bio-Dot wells were washed with TBS-T before the supernatants were added 500 µL at a time until a total of 3 mL was loaded onto the membrane. One well containing TBS-T was used as a negative control and mucin from porcine stomach (Sigma) was used as a positive control. Wells received a final wash with TBS-T before the membrane was removed and incubated with 3% acetic acid solution for 3 min followed by alcian blue pH 2.5 (Vector Labs) for 30 min at RT. The membrane was then washed with DI water and dried overnight before imaging on a ChemiDoc Imager (Bio-Rad) in grayscale. Dot blot was quantified using Fiji/ImageJ pixel density analysis.

### Image and statistical analyses

Imaris software was used to calculate Manders’ correlation coefficient for quantifying MuAstV and MUC2 colocalization from microscopy images. Fiji and ImageJ software was used for quantifying immunofluorescence microscopy images of HAstV1 infections and analyzing the percentage of overlapping fluorescence intensity signal between HAstV1 capsid and MUC2. Statistical analyses were performed in GraphPad Prism 10.

## Supporting information

S1 Fig Qualitative and quantitative analysis of goblet cells from infected C57BL/6J and immunocompromised mice. (A) Representative image of ISH with a MuAstV genome-specific probe (red) collected from C57BL/6J duodenal tissue at 6 dpi, scale = 20 μm. (B) Percent of goblet cells classified by mucus cell states (n = 5 muMT mice, n = 262 infected goblet cells, n = 300 uninfected goblet cells). (C) Percent of goblet cells classified according to virus and mucus cell states (n = 5 CD-1 Nude mice, n = 225 infected goblet cells, n = 280 uninfected goblet cells). All data are presented as mean ± SEM. Statistical testing for (B) and (C) Unpaired t-tests with Holm-Šidák method for multiple comparisons; *p < 0.05; ns, non-significant.
(TIF)

S2 Fig Effects of air-liquid interface (ALI) culture of HT29-MTX-E12 and Caco-2 cells on *MUC2* expression and HAstV1 infection. HT29-MTX-E12 cells cultured for 1 week on transwell inserts, followed by 1 additional week at ALI or no ALI. (A) *MUC2* expression normalized to *GAPDH* and relative to no ALI wells. (B) *MUC2* expression post-HAstV1 infection normalized to *GAPDH* and relative to uninfected wells with or without ALI at 24 hpi. (C) Fold change in HAstV1 genome copies quantified from the combined cells and supernatant collected from transwells at 24 hpi relative to no ALI wells. (D-F) Same as shown in A-C except HT29-MTX-E12 cells were cultured for 2 weeks on transwell inserts, followed by 1 additional week at ALI or no ALI. (G-I) Same as shown in A-C except Caco-2 cells were cultured for 1 week on transwell inserts, followed by 1 additional week at ALI or no ALI. (J-L) Same as shown in A-C except Caco-2 cells were cultured for 2 weeks on transwell inserts, followed by 1 additional week at ALI or no ALI. All data are presented as mean ± SEM. Statistical testing for (A, D, G, & J) Mann-Whitney U test, (B, E, H, & K) Kruskal-Wallis test, and (C, F, I, & L) Welch’s t-test; ****p < 0.0001, ***p < 0.001, **p < 0.01, *p < 0.05; ns, non-significant.
(TIF)

S3 Fig Negative control for mucin granule isolation and immunostaining. Mucin granules isolated from uninfected MRC-5 cells immunostained for HAstV1 capsid (red) and MUC2 (green), scale bar = 5 µm.
(TIF)

S4 Fig Positive control for FCGBP immunostaining. LS174T cells immunostained for FCGBP (green), MUC2 PTS (red), and DAPI (blue), visualized by confocal microscopy. Scale bar = 5 µm.
(TIF)

S5 Fig Single purified mucin granule fraction shows positive staining for MUC2 and HAstV1 capsid. Purified mucin granule fractions identified in (Fig 7A) were removed from sucrose and immunostained for MUC2 C-term (green) and HAstV1 capsid (red). Scale bar = 90 µm.
(TIF)

S6 Fig Effects of carbachol on HAstV1 egress in Caco-2 cells. Fold change in HAstV1 titer quantified from supernatants collected after a 1-hour incubation with 10 μM Cch with infected Caco-2 cells at 24 hpi relative to vehicle-treated cells. Data are presented as mean ± SEM. Statistical testing used Welch’s t-test; ns, non-significant.
(TIF)

S1 Data All plotted data points and uncropped blots for Figs 1–8, S1–S6. (XLSX)
